# NSL23 dataset for alphabets of Nepali sign language

**DOI:** 10.1016/j.dib.2024.110080

**Published:** 2024-01-23

**Authors:** Jhuma Sunuwar, Samarjeet Borah, Aditi Kharga

**Affiliations:** Sikkim Manipal Institute of Technology, Sikkim Manipal University, Sikkim, 737136, India

**Keywords:** Nepali sign language, NSL, NSL23, Consonant, Vowel, Sign language translation, Computer vision

## Abstract

Nepali Sign Language (NSL) is used by the Nepali-speaking community in Nepal and in Indian states such as Sikkim, the hilly region of North Bengal, some parts of Uttarakhand, Meghalaya, and Assam. It consists of the International Manual Alphabet (A-Z), Nepali consonants, vowels, conjunct letters, and numbers represented in the form of one-handed fingerspelling or Nepali manual alphabet. The standard gestures for NSL have been published by the Nepal National Federation of the Deaf & Hard of Hearing (NFDH).

To learn Nepali Sign Language, the first step is to understand its alphabet set. The use of technology can help ease the learning process. One of the application areas of computer vision is translating sign language gestures to either text or audio to facilitate communication. This is an open research area. However, NSL translation is one of the less explored research areas because there is no dataset available to work on for NSL. This paper introduces the Nepali Sign Language Dataset (NSL23), which is the first of its kind and includes vowels and consonants of the Nepali Sign Language alphabet. The dataset consists of .mov videos performed by 14 volunteers who have demonstrated 36 consonant signs and 13 vowel signs either in one full video or character by character. The dataset has been prepared under various conditions, including normal lighting, dark lighting conditions, prepared environments, unprepared environments, and real-world environments. The volunteers who performed the NSL gesture have been classified as 9 beginners who are using NSL for the first time and 5 experts who have been using NSL for 5 to 25 years. NSL23 contains 630 total videos representing 1205 gestures. The dataset can be used to train machine learning models to classify the alphabet set of NSL and further develop a sign language translator.

Specifications Table

**Table:** 

Subject	Computer Science
Specific subject area	Hand Gesture Detection, Gesture Categorization, Sign Language Detection
Type of data	Video (.mov)
How the data were acquired	Data was acquired using Nikon D3200 and Iphnoe7 Plus. Configuration for both is given below: • Nikon D3200 24.2MP Digital SLR Camera, CMOS Sensor, Video Resolution-1280×720, • Mobile phone camera Iphone7 Plus: 12 MP, f/1.8, 28 mm (wide), 1/3", PDAF, OIS, 12 MP, f/2.8, 56 mm (telephoto), 1/3.6", AF, 2x optical zoom. Video: 4K@30fps, 1080p@30/60/120fps, 720p@240fps. **Data acquisition Process** • Data were acquired in the form of video(.mov) • Each volunteer was asked to perform hand gestures facing the camera. Gesture was performed using only the right hand. • To ease cropping and extraction operation during pre-processing, the hand performing the gesture was placed beside the face. • Duration of video captured character by character varied from 1 to 5 s and video comprising all character comprises of 14.45 s to 4.5 min. **Environment**- Prepared, unprepared, **Lighting**- Bright, Dark, Normal**Location**-Indoor, Outdoor has been considered while capture the data. • Each volunteer has performed 36 Consonant and 13 vowels either as a full video or one character per video. • Repetition of data is done to generate cropped video in the case of volunteer number S1, S2 and S3. • Volunteer number S3, S4, S5, S6 and S14 have performed all characters in single shot video as they are experienced users of NSL.
Data format	Raw
Description of data collection	Data was collected from 14 volunteers, categorized as 5 Native (>25 years and >5 years of using NSL) and 9 beginners learning NSL.Dataset has 630 total videos containing 1205 gestures for 36 consonants and 13 vowels of NSL. 516 Videos for consonants that have 971 gestures, 114 videos for vowel that has 234 gestures.Data was collected in two types of environments. (1) **Prepared environment** - Black background by putting a black chart paper at the background of the wall where the gesture is performed. (2) **Unprepared environment**- No black chart paper was used to change the background while performing the gesture. Real world environment indoor and outdoor has been considered.**Lighting condition** – Natural lighting condition has been considered while capturing the data at indoor and outdoor i.e., no extra light was focused on the volunteer performing the gesture. Bright and Dark condition for indoor videos is referring the videos captured by turning the room lights and off respectively.
Data source location	• **Institution:** Sikkim Manipal Institute of Technology, Sikkim Manipal University • **City/Town/Region**: Rangpo/Majitar • **Country:** India • **Latitude and longitude:** 27°10′57.5"N 88°30′07.8"E
Data accessibility	**Repository name:** Nepali Sign Language - Consonant and Vowel **Data identification number:**10.5281/zenodo.10215687 (version1) 10.5281/zenodo.10478554 (version2) **Direct URL to data:**https://shorturl.at/lsSUW

## 1 Value of the data

• The users of Nepali Sign Language will greatly benefit out of this dataset as it is first of its kind and will be used by researchers to develop automatic Nepali sign language recognition application. There are many existing datasets for other languages [2], [3], [4], [5],^7^,^8^,[10], [11], [12], [13],^15^,^17^,[19], [20], [21], [22], [23]
• This dataset can be used to develop Nepali sign language translator a mean of communication between the deaf and non-deaf communities.
• This dataset will help researchers and developers to train and test machine learning, deep learning models [3,^4^,18] created specifically for automated Nepali Sign Language hand signs detection and recognition.
• The Nepali sign language dataset opens up a new avenue for future study and development of real-world gesture recognition for researchers as it comprises of real-world condition [3,^6^,8] where varying lighting conditions, backgrounds, and hand positioning has been considered.
• This will benefit Nepali speakers who want to learn Nepali Sign Language as well as those developing translation applications to serve as a communication tool between NSL user and non-user [1,^14^,19].
• It can be used to create new similar dataset or to expand the dataset by replicating the samples or adding new samples of Nepali sing language in different background conditions, lighting conditions, and orientation to further improve NSL23 dataset.

## 2 Background

Several hand gesture datasets are available for public use [2], [3], [4], [5],^7^,^8^,[10], [11], [12], [13],^15^,^17^,[19], [20], [21], [22], [23]. However, most of them lack variations in the gestures they provide. While some sign language datasets that are publicly available offer a collection of frames to represent a gesture, others provide videos that were collected under the same environmental and lighting conditions. The paper aims to create a comprehensive dataset that includes various lighting conditions such as indoor, outdoor, bright, dark, and natural lighting. The dataset will also consider prepared and unprepared backgrounds. The scenario for the dataset involves volunteers performing gestures at different positions and heights in a real-world setting. The objectives of the dataset are to:

• To create a dataset that provides a Nepali Sign Language alphabet set (Consonant and Vowel) and make it available publicly.
• To provide a dataset having variations in environments and lighting conditions.
• To provide the dataset in raw form so that users can preprocess the data as per their requirement and use it to train supervised or semi-supervised or test the supervised and unsupervised machine learning model and deep learning models [6,^9^,^10^,^16^,18].
• To encourage researchers to start working in Nepali Sign Language translation.

## 3 Data Description

The NSL23 dataset contains two folders: one for consonants and one for vowels. Each folder contains subfolder which further contains videos. The details of both folders are provided in Tables 1 and 2 respectively.

**Table 1:** Description of NSL23 Consonant Dataset.

Sl No	Name of the folder	No. of videos	Description
1	S1_NSL_Consonant_Dark	36	Indoor unprepared in dark condition captured using phone camera
2	S1_NSL_Consonant_Bright	36	Indoor unprepared in normal condition captured using phone camera
3	S1_NSL_Consonant_Dark_Cropped	36	Indoor unprepared in dark condition captured using phone camera
4	S1_NSL_Consonant_Bright_Cropped	36	Indoor unprepared in normal condition captured using phone camera
5	S2_NSL_Consonant_Dark	36	Indoor unprepared in dark condition captured using phone camera
6	S2_NSL_Consonant_Bright	36	Indoor unprepared in normal condition captured using phone camera
7	S2_NSL_Consonant_Dark_Cropped	36	Indoor unprepared in dark condition, preprocessed video captured using phone camera
8	S2_NSL_Consonant_Bright_Cropped	36	Indoor unprepared in normal condition, preprocessed video captured using phone camera
9	S3_NSL_Consonant_Prepared	2 [2×36=72 gestures]	2 indoor prepared black background captured using camera and phone camera
10	S3_NSL_Consonant_Prepared_Cropped	36	Indoor prepared black background captured using phone camera
11	S3_NSL_Consonant_Real_World	2 [2×36=72gestures]	2 outdoor unprepared environment-real worlds captured using phone camera
12	S4_NSL_Consonant_Prepared	2 [2×36=72 gestures]	Indoor prepared black background taken from mobile camera and phone camera in slightly different angle.
13	S5_NSL_Consonant_Prepared	1 [36 gesture]	Indoor prepared black background captured using camera
14	S6_NSL_Consonant_Prepared	2 [2×36=72 gestures]	Indoor prepared black background taken from mobile camera and phone camera in slightly different angle
15	S6_NSL_Consonant_RealWorld	1 [36 gestures]	Indoor unprepared environment captured using phone camera
16	S7_NSL_Consonant_Prepared	36	Indoor prepared black background captured using phone camera
17	S8_NSL_Consonant_Prepared	36	Indoor prepared black background captured using phone camera
16	S9_NSL_Consonant_Prepared	1 [36 gestures]	Indoor prepared black background captured using phone camera
17	S10_NSL_Consonant_Prepared	36	Indoor prepared black background captured using phone camera
18	S11_NSL_Consonant_Prepared	36	Indoor prepared black background captured using phone camera
19	S12_NSL_Consonant_Prepared	35	Indoor prepared black background captured using phone camera
20	S13_NSL_Consonant_Prepared	1 [36 gestures]	Indoor prepared black background captured using phone camera
21	S14_NSL_Consonant_RealWorld	1 [36 gestures]	Indoor unprepared background captured using phone camera

**Table 2:** Description of NSL23 Vowel Dataset.

Sl No	Name of the folder	No. of videos	Description
1	S1_NSL_Vowel_Unprepared_Bright	13	Indoor Unprepared Background captured using phone camera
2	S1_NSL_Vowel_Unprepared_Bright_Cropped	13	Indoor Unprepared Background captured using phone camera
3	S1_NSL_Vowel_Unprepared_Dark	13	Indoor Unprepared Background captured using phone camera
4	S1_NSL_Vowel_Unprepared_Dark_Cropped	13	Indoor Unprepared Background captured using phone camera
5	S2_NSL_Vowel_Unprepared_Bright	13	Indoor Unprepared Background captured using phone camera
6	S2_NSL_Vowel_Unprepared_Bright_Cropped	13	Indoor Unprepared Background captured using phone camera
7	S2_NSL_Vowel_Unprepared_Dark	13	Indoor Unprepared Background captured using phone camera
8	S2_NSL_Vowel_Unprepared_Dark_Cropped	13	Indoor Unprepared Background captured using phone camera
9	S3_NSL_Vowel_Prepared	2 [2×13=26 gestures]	Indoor Prepared Background captured using camera and phone camera
10	S3_NSL_Vowel_Unprepared_RealWorld	1 [13 gesture]	Outdoor Unprepared Background captured using phone camera
11	S4_NSL_Vowel_Prepared	2 [2×13=26 gestures]	Indoor Prepared Background captured using camera and phone camera
12	S5_NSL_Vowel_Prepared	1 [13 gestures]	Indoor Prepared Background captured using camera
13	S6_NSL_Vowel_Prepared	2 [2×13 =26 gestures]	Indoor Prepared Background captured using camera and phone camera
14	S6_NSL_Vowel_Unprepared	1 [13 gestures]	Indoor Unprepared Background captured using phone camera
15	S14_NSL_Vowel_Unprepared	1 [13 gestures]	Indoor Unprepared Background captured using phone camera

Table 1 shows the organization of subfolders which contains 36 consonant of Nepali sign language.

Table 2 shows the organization of subfolders which contains 13 vowels of Nepali sign language.

Tables 3 and 4 to identify each video alphabetically. The direction of the arrow in the hand gesture represents the direction of movement of the hand while performing the dynamic gesture. Volunteers were given the table to understand and learn the gesture before capturing their video as shown in Fig. 3.

**Table 3:** Nomenclature used for consonant videos (All Static Gesture).

**Table 4:** Nomenclature used for vowel videos (Static and Dynamic gesture).

The dataset has been made publically available for use in Zenodo platform.Using the link : https://shorturl.at/lsSUW, user will be able to download the following files:

$$\begin{array}{c} i.\;\text{Details}\;\text{Dataset}.\text{docx}-\text{Gives}\;\text{description}\;\text{table}\;\text{for}\;\text{each}\;\text{video}\\ \begin{array}{c} \text{ii}.\;\text{NSL}\_\text{Consonant}\_\text{Part}\_1.\text{zip}\\ \text{iii}.\;\text{NSL}\_\text{Consonant}\_\text{Part}\_2.\text{zip}\\ \text{iv}.\;\text{NSL}\_\text{Consonant}\_\text{Part}\_3.\text{zip} \end{array}\}\\ v.\;\text{NSL}\_\text{Vowel}.\text{zip} \end{array}\text{NSL}\_\text{Consonant}$$

Each folder inside NSL_Consonant and NSL_vowel has been labeled using the naming convention as follows: volunteer number (S1 to S14) _ NSL _ Consonant (or Vowel) _Bright (or Dark, Prepared, Unprepared, RealWorld) _Cropped (if cropped video). Each folder consists of videos in which volunteers have demonstrate the hand gestures for consonant and vowel alphabets of NSL respectively. These videos are named using the naming convention specified in Table 3 and Table 4. If a video displays one character of NSL consonant/Vowel at a time, otherwise the word "all" is used to signify that the video contains all the alphabet of NSL consonant/vowel in a single video. Table 1 and Table 2 provide the detail content of NSL_Consonant and NSL_Vowel respectively. Users can follow the above given naming convention to access the corresponding data as per requirement.

## 4 Experimental Design, Materials, and Methods

**Fig. 1:**
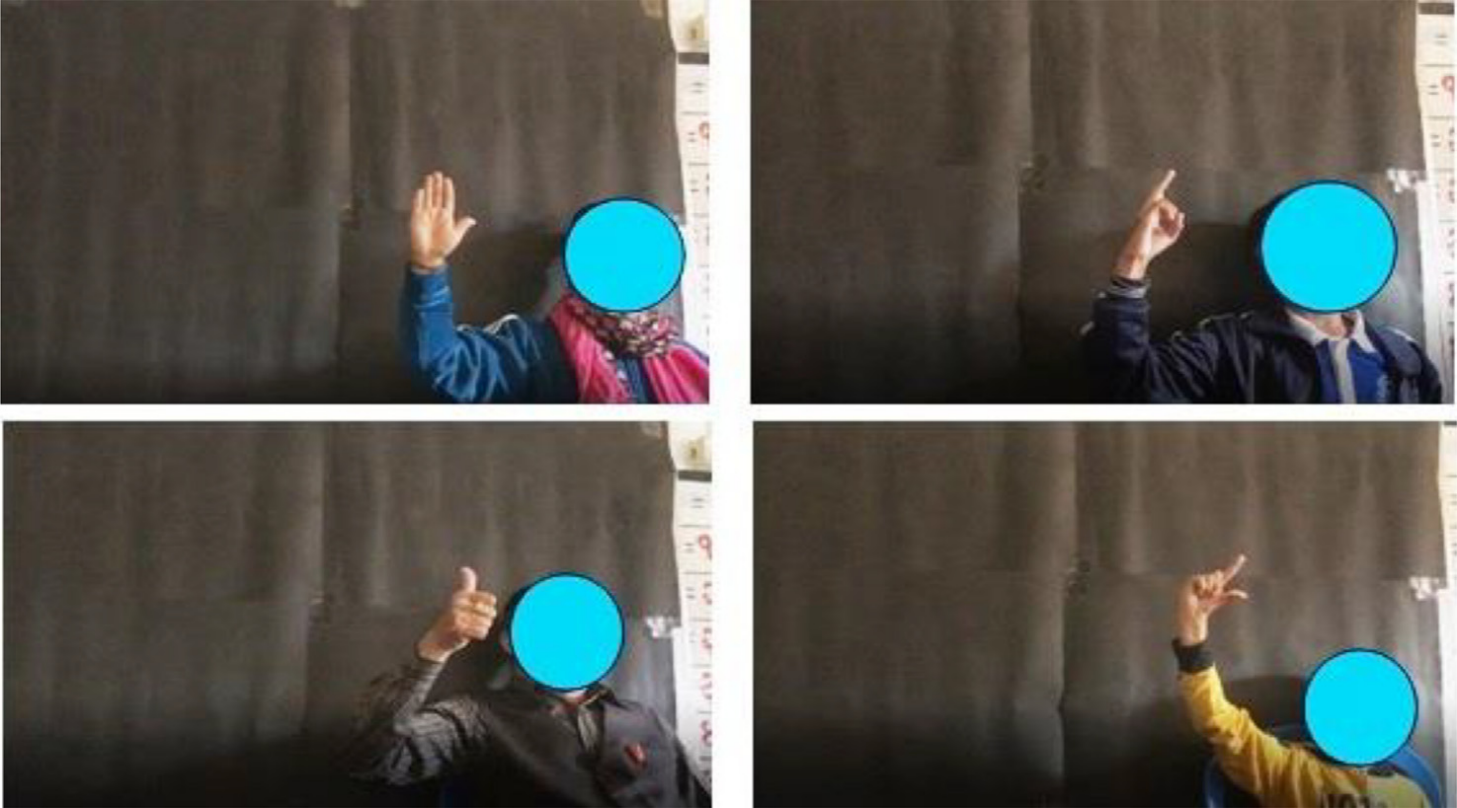
Volunteers posing for NSL23 dataset in prepared environment.

To learn any sign language [2], [3], [4],^7^,^20^,22], one must start with its alphabets. This paper focuses on the construction of Nepali Sign Language alphabets (consonant and vowel) and has been named NSL23.To start with the data set preparation that can be used by Machine learning model to recognize real world NSL gesture, training data needs to be prepared. A faculty of a special school was approached to assist with the data collection process. He teaches Nepali sign language to differently able students of class 1 to class 5. With his support it was possible to collect raw data in the form of video in a prepared and un-prepared environment. The prepared environment was created by pasting black chart paper at the background while the volunteer performing the gesture pose facing the camera kept in front of them. Fig. 1 shows some volunteers and the environment used during data collection. Total there are 14 volunteers out of whom 5 volunteers are native users of Nepali sign language and are either the faculty using NSL or students who have been using it for more than 5 years.

**Fig. 2:**
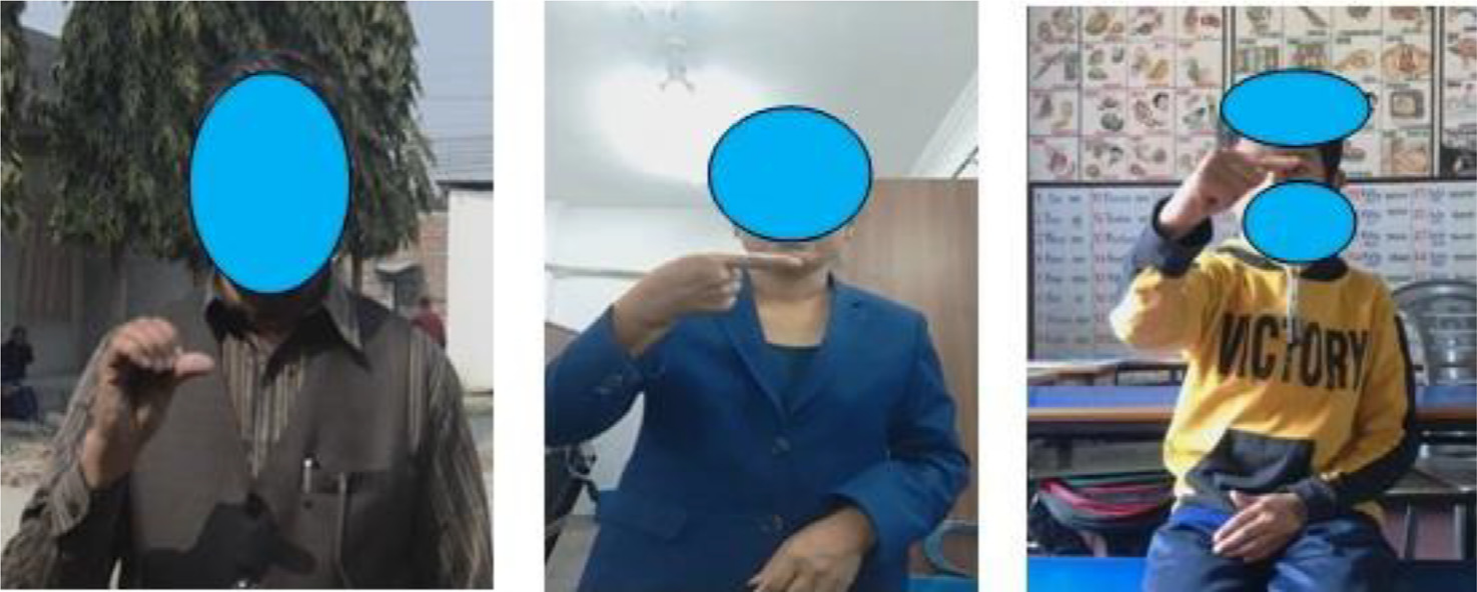
Outdoor and Indoor Environment, Unprepared Environment to Showcase Real World Scenario.

Data has been captured in first persons’ view [8] which means the camera is placed in front of the volunteer performing the gesture. To increase the scope of the dataset various environmental and illumination conditions have been considered. The videos are taken indoor- prepared as shown in Fig. 1, unprepared, and outdoor to make it more dynamic. Fig. 2 show the different real world environment considered while acquiring the data.

**Fig. 3:**
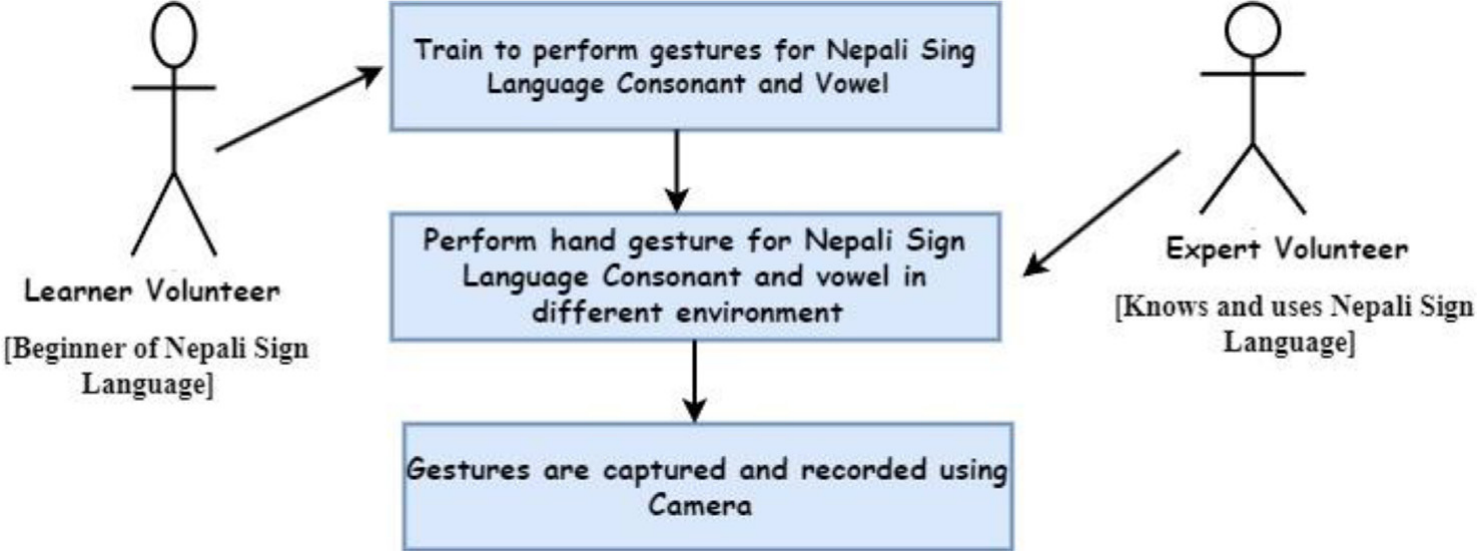
Process prior to data generation.

Initial phase of collecting NSL23 dataset comprises of 630 total videos, 1205 gestures performed by 14 volunteers labeled as S1 to S14 to identify each volunteer. Volunteers S3, S4, S5, S6, and S14 are experts in NSL, so they provided consonant and vowel gestures in one take. However, the remaining volunteers were new to NSL, so their signs had to be captured character by character as shown in Fig. 3. A prepared environment was created by putting black chart paper in the background to ease the segmentation process of separating foreground and background. For the dataset, outdoor videos were shot in a natural light environment, while indoor videos was shot with natural light as well as by turning on room light. To illustrate the illumination condition, all the videos have been labeled as dark or bright depending on the lighting environment used during the video shoot. To increase variation in the dataset, some videos were preprocessed using Adobe Express tools to crop and keep only the hand portion. These videos are labeled as ``cropped''.

**Fig. 4:**
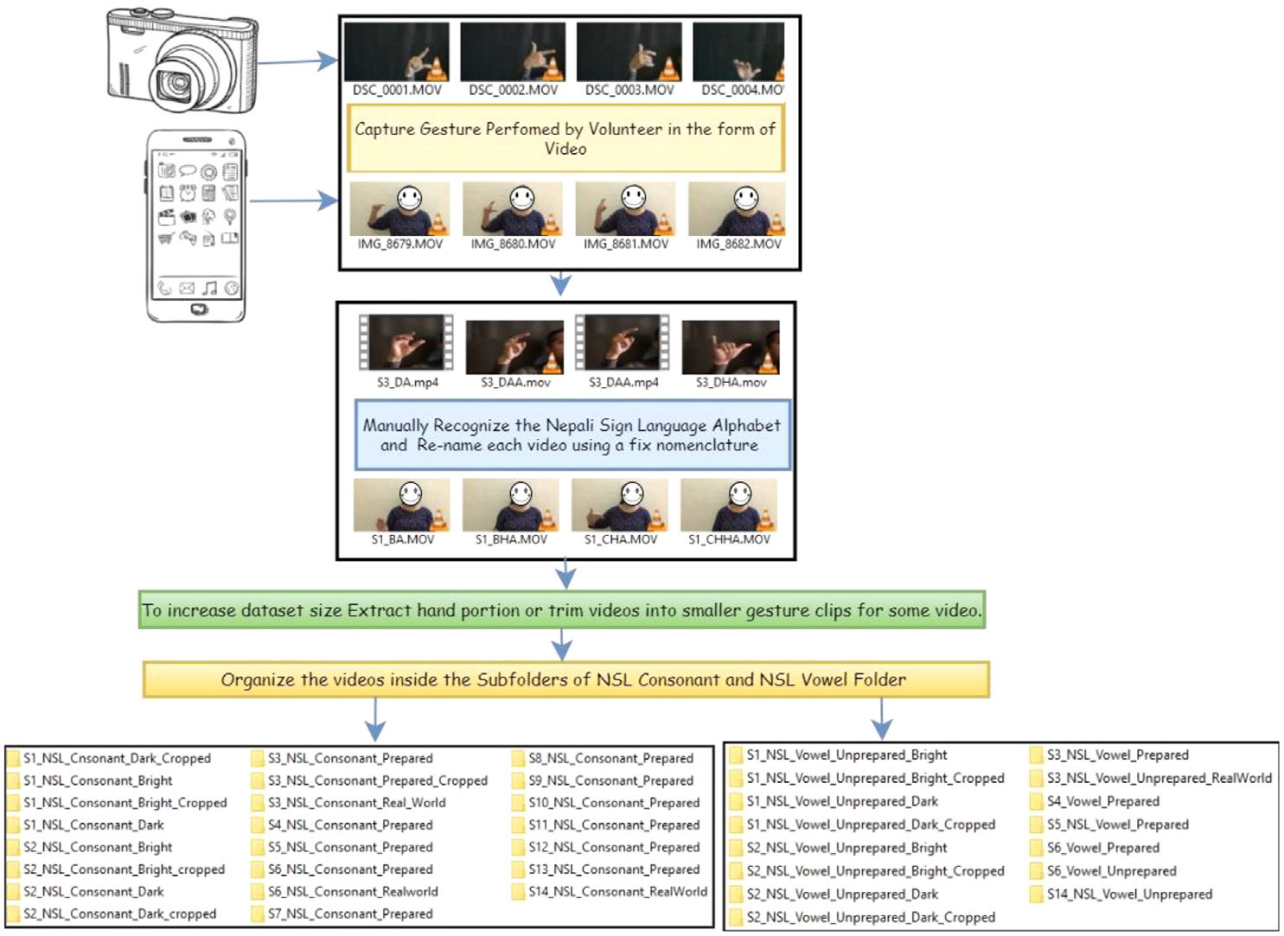
Data structuring and labeling process.

The methodology used for the collecting and structuring of datasets is shown in Figs. 3 and 4.

The dataset was captured using the process shown in Fig. 3 and structured using the methodology illustrated in Fig. 4.

To curate the dataset, we had to identify which videos to include or discard; label each included video, and structure it, which required human intervention. This process was time-consuming. To label each video nomenclature given in Table 3 and Table 4 has been used.

**Fig. 5:**
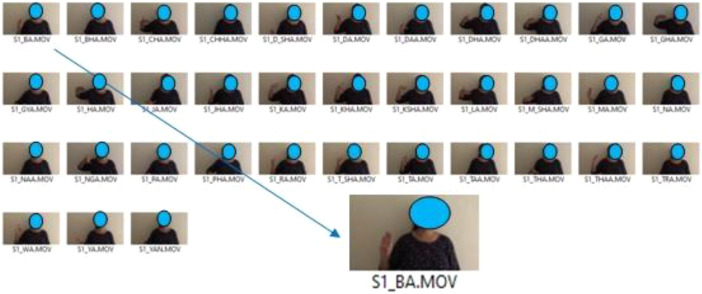
Indoor Unprepared Background (Dark Condition).

**Fig. 6:**
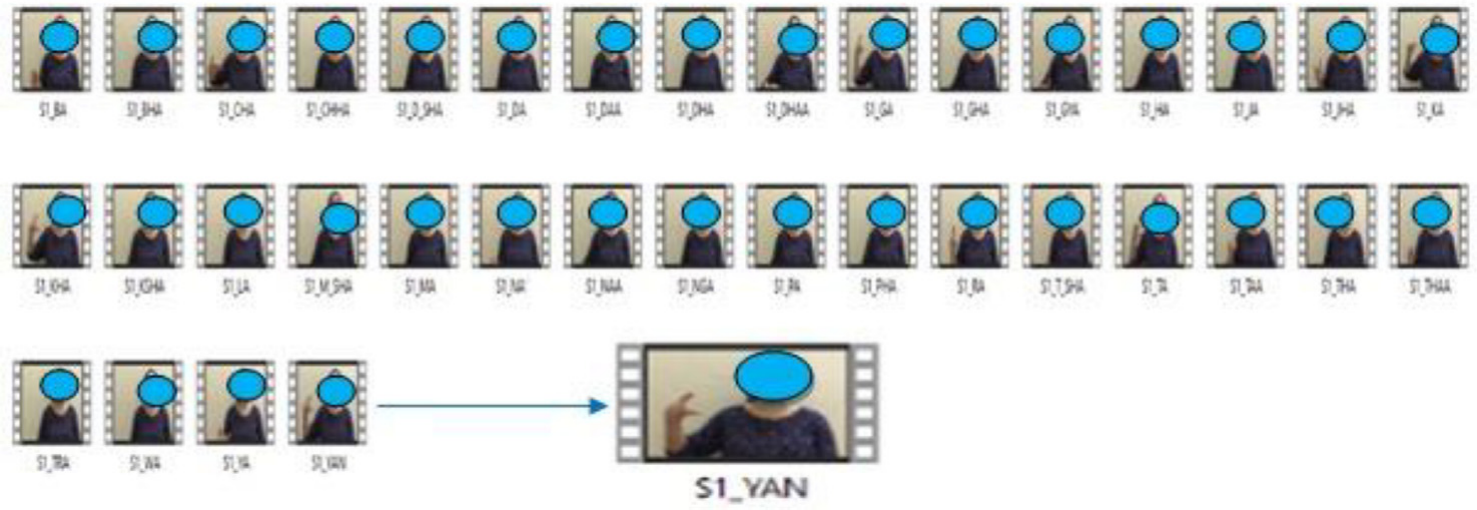
Indoor Unprepared Background (Bright lighted condition).

Fig. 5, Fig. 6 shows the different lighting conditions used in dataset.

**Fig. 7:**
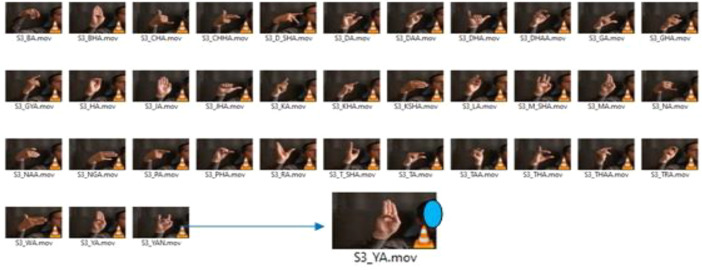
Indoor Prepared Black Background and cropped.

Video containing incomplete or wrong gesture was manually discarded. The videos were trimmed and cropped using the Adobe Express (online video editing website) [source: https://www.adobe.com/express] as shown in Fig. 7 for videos labeled as cropped. Other preprocessing approach has not been performed in NSL23 to encourage users apply preprocessing techniques as per their need to make the dataset suitable for their purpose.

## Ethics Statements

This data collection work has involvement of human subjects. Consents from all the subjects/participants are taken in writing form and scanned copy of the same is attached as supplementary material with this submission.

## CRediT authorship contribution statement

**Jhuma Sunuwar:** Data curation, Formal analysis, Writing – original draft. **Samarjeet Borah:** Conceptualization, Project administration, Supervision, Writing – review & editing. **Aditi Kharga:** Data curation.

## Data Availability

Nepali Sign Language -Consonant and Vowel (Original data) (zenodo.org)
